# Some New Methods in Skin Grafting

**Published:** 1906-09

**Authors:** John Egerton Cannaday

**Affiliations:** Surgeon-In-Charge Sheltering Arms Hospital, Hansford, W. Va.


### SOME NEW METHODS IN SKIN GRAFTING.*

BY JOHN EGERTON CANNADAY, M.D.,

Surgeon-In-Charge Sheltering Arms Hospital, Hansford, W. Va.

I have selected my subject from the domain of minor surgery for the reason that it should appeal to the general practitioner, who will not infrequently have occasion to do such work. I have several times had occasion to treat old ulcerated surfaces in which epidermization had for months made absolutely no advance, and

*Read before the Central Tri-State Medical Society, of West Virginia, Kentucky and Ohio, July 19, 1906, at Clyffeside, Park, Ky.

yet the physician in charge had never made an attempt at transplantation. In many forms of minor surgical procedure some degree of satisfaction in results is often obtainable even when the methods are careless. In the particular field of this subject, however, a technique absolutely careful in all the minutiæ of its application is necessary to the achievement of success.

The need of epithelial grafting arises from several causes, among which are: to cover defects in skin integrity caused by the excision of malignant growth, deep burns, lacerated wounds in which sufficient flaps for covering can not be secured, the sloughing of flaps, gangrene due to frostbite and other causes, the correction of deformities, varicose and other ulcers, destructive skin lesion, as lupus, blastomycosis, etc. We know that such bared areas can cover themselves only by the reproduction of epithelium around the edges, that after a varying length of time dependent on the individual case this border-line epithelium becomes exhausted and ceases to further divide and reproduce itself. It is then we must assist nature. Indeed, it is not well to wait until that stage arrives.

In the choice of material, unless the area to be grafted is large, it is usually most convenient to get the grafts from the individual needing them, unless some self-sacrificing individual is at hand to donate the necessary integument. One can often get suitable material from amputations and other operative procedures without robbing the patient of his own integument, inasmuch as molecular life is maintained for some hours after somatic death. The skin of the white, when grafted on the negro will, in the course of time, become like that of its host, and vice versa. Frogs, chickens, pigs, and other animals have contributed the covering of man with a very good grace. On one occasion I grafted the skin of a water lizard, commonly known as a "water-dog," onto the foot of an Italian with a fair degree of success. The skin of the arm or thigh is of a suitable character for this work, and from its situation is quite accessible. When the defect is on a limb it is well usually to take grafts from a nearby location.

Formerly all skin defects were covered by plastic methods, the sliding and transference of flaps. Later it was found by Thiersch that a new vascular supply had been formed in grafts eighteen hours old.

Wolf, Hirschberg and Krause devised methods by which the entire thickness of the skin is transferred. Reverdin planted infinitesimal islands of epithelium on raw surfaces.

Thiersch avoided the extremes of both methods and used strips of considerable size and length, but involving only a part of the thickness of the skin.

These grafts not only take well, but the injury caused by their removal heals readily, owing to the deeper layers of the skin being left behind.

Czerny and Folfler originated the successful transplantation of mucous membrane. The former devoting his attention to the conjunctiva and the latter doing work on mucous membranes in general. The transplantation of mucous membranes is more difficult than skin, and necessitates a more refined technique.

Thiersch taught that the clean, healthy granulations should be curetted, the oozing blood controlled by sponge pressure, and the grafts applied.

Isnardi applied grafts directly to the granulations without disturbing or removing them, and holds them in place with thin gauze tightly stretched over the grafts. He attempts by frequent dressings, alternately dry and wet, to secure rosy-red, hard, healthy granulations; to prevent too great a tendency to bleeding he touches the granulations with a thermocautery, if necessary.

Bruning and Sneve use no retentive dressing, but expose the grafted surface to the open air. This method has much, especially in the way of simplicity, to commend it, and can be used in many cases.

Buller recommends the use of narrow strips of isinglass plaster to securely retain the grafts in place.

Wilcox cleanses infected areas by the use of peroride of green soap, followed by the application of a one per cent. formaldehyd solution, which is allowed to remain for several hours. He curettes away the granulations, controls oozing by the direct application of the Esmarch bandage over the bleeding surface, and then applies his grafts.

Grafting is often done for the restoration of the eyelids for cosmetic and functional purposes, and presents quite a number of difficulties.

The operative field is movable and uneven. The graft on the upper lid must be light, so as not to press too heavily on the ball or to interfere with the mobility of the lid. The elastic tissue of the eyelids constitutes a surface that will cause a graft when placed thereon to shrink. Dodd, who has investigated this line of work thoroughly, uses Wolfe's grafts where the entire thickness of the lid is destroyed. In other cases, Thiersch's. He secures immobility in the lower lid by suturing it to the upper, and in case the upper lid is alone to be treated, stitches it to adhesive strips attached to the cheek.

Campbell, of London, finds that by the use of grafts to fill in skin defects caused by the removal of keloid lupus, and sarcoma, rather than by suture approximation the tendencies to epithelial recurrence, is considerably lessened. While for this minor opertive procedure an anesthetic is not always necessary, yet it is in most cases desirable.

Among the various means used to secure a clean, healthy granulating surface a wet bidaily dressing of gauze wet with normal saline solution or a weak solution of chloral hydrate (grs. ii to the oz. of water) covered with oiled silk or other impervious material, is probably as effective as anything.

In my own practice I use the Thiersch or Shaving graft. After securing a clean, healthy granulation site I have the part from which the flaps are to be taken carefully cleansed in the usual manner preliminary to operative work. I wash the granulations with hot saline solution, evenly freshen the surface by a gentle rubbing with a new sterile Tampico fibre nail-brush, which will freshen the granulations without destroying them. I long ago abandoned the curette as being a very unsatisfactory instrument for this sort of work. It tears off the granulations entire, leaves an irregular surface which has not nearly so good vascular supply as the granular surface.

Lauenstein has recently suggested rubbing with a gauze pad to freshen the surface. I tried his method in several cases recently. I found it quite effective where the granulations were tender, but in one case where the granulations were firm and hard, I had to return to the use of the nail-brush. A few moments pressure with a sponge wet with hot saline solution to check the oozing of blood and the part is ready for the reception of the grafts. The razor should be sharp. An instrument having a metal handle that can be boiled is preferable, but the ordinary razor carefully washed with soap and hot water, then with alcohol will suffice. The part from which the graft is to be taken is exposed, the skin made tense and bulging by grasping with the hand, or by the use of sharp retractors. Grafts half the thickness of the skin, half an inch wide and one and one-half inches long, are suitable for most cases. If the skin is held properly these can be easily sliced off by sawing movements of the razor. As each graft is severed turn it over the left index finger, skin side next to the finger, carry graft direct to the denuded site, press it down on the surface with the finger, with a small probe smooth and straighten it out as you wish it to remain and repeat the process until the surface to be grafted is covered. By transferring each each skin strip directly you will be able to cut them in proper size, shape and number, the proportion of takes will be larger. I have always had the surface from which the grafts are taken dry, and transferred them without the intervention of anf saline or other solution, which only serves the purpose of rendering a delicate piece of work more difficult and of diminishing the chances of success.

Cover the grafted site with thin dental rubber dam, one piece if the area is small or, strips, criss-crossed for drainage, if the area is large. Some recommend cargile membrane, some green protective and others, more or less impervious substances. I place a small gauze pad over this, securely anchor it from all sides with narrow strips of zinc oxide adhesive to prevent displacement of the grafts, apply externally a cotton, gauze and bandage dressing.

In many cases it will be well to encase the part with plaster of Paris to secure immobility. A slight amount of pressure will keep the grafts in apposition with the part, will prevent serum from collecting between graft and wound and separating them, and will control bleeding. On the other hand, too much pressure will destroy every vestige of life in the Thiersch flaps and render the pains of the operator for naught.

Formerly we were taught to keep the dressings wet with the physiologic saline solution. It was so valuable for some things it was supposed to be for everything. I discontinued it as being totally devoid of value and began the use of sterile vaseline or petrolatum as a lubricant to prevent the dressing from becoming adherent.

Parmenter suggested the use of sterilized oil instead of the mussy saline dressing. For some time I have used the plain dry dressing as described above without any cause for regret. It possesses the advantages of simplicity and neatness, it renders the after-care less complicated, and can easily be removed by the free use of peroride of hydrogen and sterile water without disturbing the underlying epithelium in the least. I practically never remove the dressing until a week has elapsed, and the wound usually requires but little care after that.

I have practiced the method described in twenty-three cases of skin-grafting without a failure. I have used it chiefly in the repair of defects caused by burns, and by sloughs from lacerated and contused wounds.

In conclusion, I would emphasize the value of the direct transference of grafts, the use of the nail-brush, instead of the curette, and the simplicity and effectiveness of the dry dressing moored in placed by adhesive strips.

---

IN ingrowing toenail, evulsion of the nail gives temporary relief, but it does not cure. When the nail grows out again the condition recurs, often in aggravated form.— *American Journal of Surgery.*

